# Triglyceride to high-density lipoprotein cholesterol ratio is associated with increased mortality in older patients on peritoneal dialysis

**DOI:** 10.1186/s12944-019-1147-8

**Published:** 2019-11-15

**Authors:** Xiaojiang Zhan, Mei Yang, Ruitong Zhou, Xin Wei, Yanbing Chen, Qinkai Chen

**Affiliations:** Department of Nephrology, The First Affiliated Hospital of Nanchang University, 17# Yongwai Street, Nanchang, 330006 China

**Keywords:** Triglyceride/high-density lipoprotein cholesterol (TG/HDL-C) ratio, Mortality, Cardiovascular, Older, Peritoneal dialysis

## Abstract

**Background:**

The triglyceride (TG) to high-density lipoprotein cholesterol (HDL-C) ratio (TG/HDL-C) has been suggested as a simple method to identify unfavorable cardiovascular (CV) outcomes in the general population. The aim of this study was to investigate the association between the TG/HDL-C ratio and all-cause and CV mortality in peritoneal dialysis (PD) patients.

**Methods:**

We retrospectively analyzed patients on PD from November 1, 2005, to February 28, 2017, with a follow-up period lasting until May 31, 2017. The main outcomes were all-cause and CV mortality.

**Results:**

Among the 973 PD patients, the mean age was 49.67 ± 14.58 (y). During a median follow-up period of 27.2 months (IQR = 13.4–41.5 months), 229 (23.5%) patients died, with 120 (12.3%) dying as a result of CV diseases. The median serum TG/HDL-C ratio was 1.11 (IQR = 0.71–1.80). In a multivariate Cox regression analysis, patients with higher TG/HDL-C ratio levels (tertile 3) had a higher incidence of CV mortality (adjusted HR = 2.12; 95% CI: 1.21–3.72; *P* = 0.009) and all-cause mortality (adjusted HR = 2.08; 95% CI: 1.37–3.14; *P* = 0.001) compared to patients in tertile 1. These associations persisted after excluding the patients who have already taken lipid-lowering medications. For older patients (> 60 years), each 1-unit higher baseline TG/HDL-C level was associated with a 48% (95% CI: 1.06–2.07; *P* = 0.021) increased risk of all-cause mortality and a 59% (95% CI: 1.03–2.45; *P* = 0.038) increased risk of CV mortality; however, this association was not observed in patients ≤60 years of age.

**Conclusions:**

A higher serum TG/HDL-C ratio was an independent predictor of all-cause and CV mortality in PD patients. Furthermore, an elevated TG/HDL-C ratio was significantly associated with higher all-cause and CV mortality in older PD patients.

## Introduction

Peritoneal dialysis (PD) is a primary treatment modality for end-stage renal disease (ESRD) patients [1]. In 2014, 55,373 patients received PD in China [2]. Despite advancements in dialysis treatment technology, ESRD patients continue to experience a lower quality of life, high hospitalization rates, and high annual mortality rates of approximately 20%, a rate worse than that of many cancers [3]. A major cause of mortality is cardiovascular disease (CVD) [2], which is 10–30 fold higher in ESRD patients than in the general population [4]. Traditional CVD risk factors, such as hypercholesterolemia and obesity, have not been able to fully explain the increased mortality observed in ESRD patients. In contrast, some reports in the literature have shown that these factors are associated with better survival in dialysis patients [5–7]. However, uremic dyslipidemia in PD patients, including high levels of triglycerides (TG) and lower high-density lipoprotein cholesterol (HDL-C), could lead to an increased risk of CVD-related mortality [8].

Recently, parameters associated with inflammation, oxidative stress, insulin resistance, and endothelial dysfunction have been reported to be better at predicting CVD outcomes [9]. The TG/HDL-C ratio, which is a simple and reproducible parameter that can easily be calculated daily and is known as an atherogenic index of plasma [10], is one such parameter [11]. Accumulated evidence has shown a predictive role for the TG/HDL-C ratio in several disorders, such as fatal and nonfatal cardiovascular (CV) events [10], coronary atherosclerosis [12, 13], impaired heart-rate recovery after exercise [14], ischemic heart disease [15], CVD and coronary heart disease [16, 17]. However, dyslipidemia and its association with CVD and mortality in patients on dialysis present a unique challenge in clinical practice, as their effect on outcomes remains to be fully clarified. Recently, a large retrospective study enrolled 50,673 hemodialysis (HD) patients and showed that a higher TG/HDL-C ratio was associated with better CV and overall survival [3]. In contrast, Chen et al. [16] indicated that a higher TG/HDL-C ratio was associated with a higher incidence of CV and all-cause mortality. The difference between the two studies above may be due to the different types of patients enrolled; the study of Chen et al. [16] enrolled both HD and PD patients.

There is a paucity of data on whether the TG/HDL-C ratio is an independent predictor of mortality in PD patients. Thus far, to the best of our knowledge, only one study has indicated that a higher serum TG/HDL-C ratio is associated with an increased risk of all-cause and CV mortality in PD patients [18]. In contrast, one recent study indicated that the TG/HDL-C ratio was negatively associated with all-cause mortality among elderly adults in the general population [19]. While accumulating evidence supports the predictive power of the TG/HDL-C ratio in general and in certain subgroups, very few studies have focused on the age-related differences between the TG/HDL-C ratio and mortality in PD patients.

The aim of this study was to find an association between the TG/HDL-C ratio and all-cause and CV mortality in PD patients; furthermore, we tried to find age-related differences between the TG/HDL-C ratio and mortality in PD patients.

## Methods

### Study population and data source

**Fig. 1:**
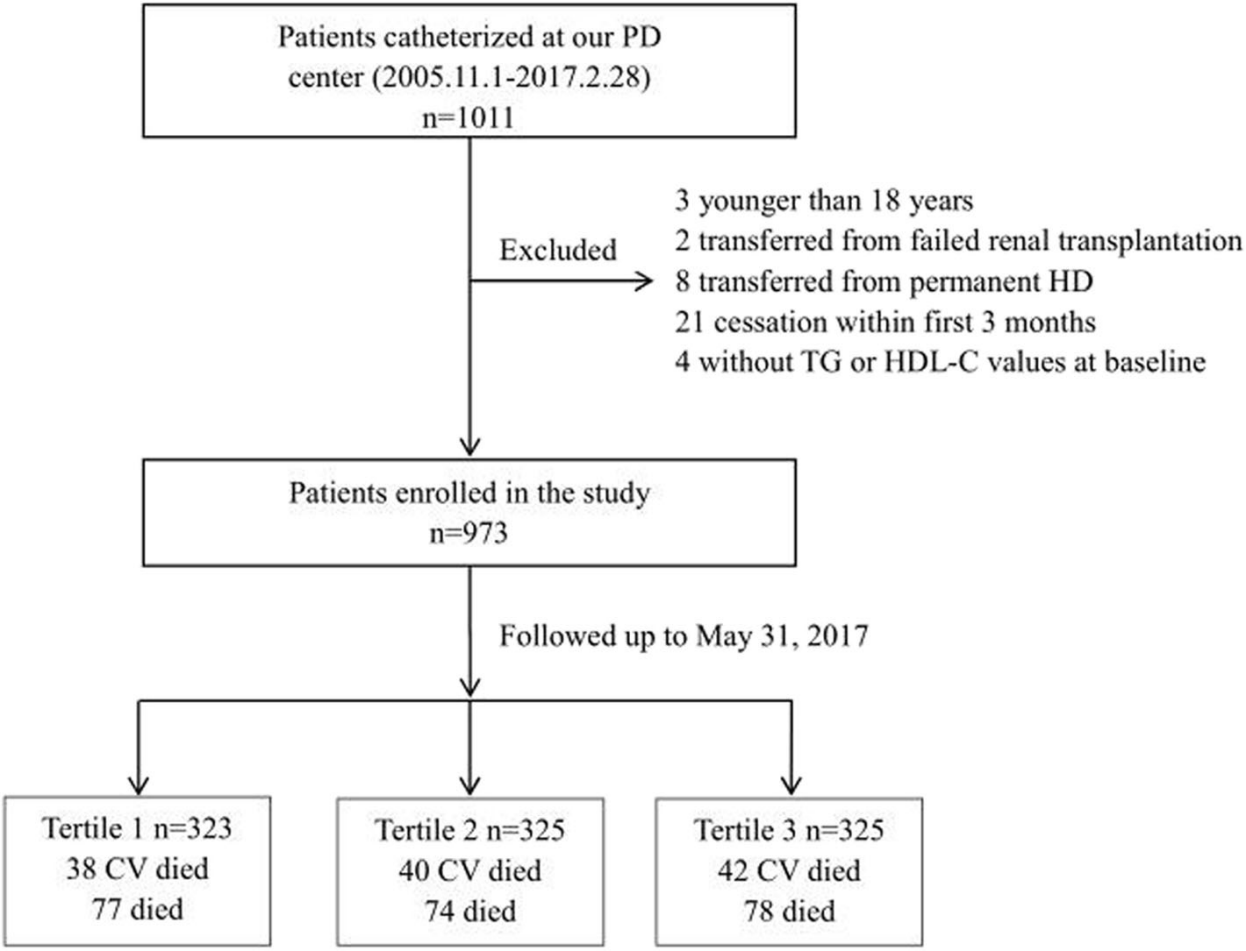
Flow chart of this study. PD, peritoneal dialysis; HD, hemodialysis; TG, triglyceride; HDL-C, high density lipoprotein cholesterol; CV, cardiovascular

The study cohort was comprised of all patients with ESRD who initiated PD between November 1, 2005, and February 28, 2017, in the PD center of The First Affiliated Hospital, Nanchang University, Jiangxi, China. The study enrolled patients who were ≥ 18 years old, were had been treated with PD therapy only in our PD center for at least 90 days, and had serum TG and HDL-C levels measured during the first 3-months of PD therapy. We excluded patients who were catheterized in other hospitals or transferred from permanent HD or who had experienced failed renal transplantation. Overall, as shown in Fig. 1, a total of 973 patients were enrolled in this study. The TG/HDL-C ratio was treated as a categorical variable and divided into tertiles (Ts): T1 (≤0.833, *n* = 323), T2 (> 0.833 to ≤1.496, *n* = 325) and T3 (> 1.496, n = 325). The reference TG/HDL-C ratio category for all analyses was T1. All study procedures complied with the ethical guidelines of the Declaration of Helsinki and were approved by the Human Ethics Committees. Written informed consent was obtained from all the patients before study enrollment.

All patients were followed up until death, cessation of PD, or May 31, 2017. All baseline data were collected within a 3-month period, starting from the date of first PD therapy. Baseline demographic data included age, sex, primary cause of end-stage renal disease (ESRD), and presence of diabetes and CVD. Clinical and biochemical data included body mass index (BMI), blood pressure, estimated glomerular filtration rate (eGFR), medication use, hemoglobin, white blood cells, serum albumin, serum uric acid, total cholesterol (CHOL), TG, HDL-C and the Charlson comorbidity index (CCI). Baseline residual renal function was assessed by eGFR using the Chronic Kidney Disease Epidemiology Collaboration creatinine equation. The CCI has been used to stratify patients in order to control for the confounding influence of comorbid conditions on overall survival and the score was calculated for each patient as the total of the patient’s comorbid conditions which have been weighted. Conditions with a weight of one included: myocardial infarction, congestive heart failure, peripheral vascular disease, cerebrovascular disease, dementia, chronic pulmonary disease, connective tissue disease, ulcer disease, mild liver disease and diabetes. Conditions with a weight of two included: hemiplegia, moderate or severe renal disease, diabetes with end organ damage and any malignancy. Moderate or severe liver disease (e.g., cirrhosis with ascites) was given a weight of 3 and metastatic solid tumor or AIDS received a weight of 6 [20]. The TG/HDL-C ratio was calculated as TG divided by HDL-C (expressed in mmol/L).

### Study outcomes

The primary outcomes were all-cause and CV mortality. Cardiovascular events that contributed to CV mortality were defined by the first occurrence of myocardial infarction, stroke, heart failure, hospitalization for unstable angina, peripheral vascular event, sudden death, death associated with a cardiovascular procedure, or death due to aneurysm dissection or rupture, fatal pulmonary embolism, or death due to other or unknown cardiovascular causes [21].

### Statistical analysis

All statistical analyses were performed using SPSS software version 21.0 (SPSS Inc., Chicago, IL). A *P* value < 0.05 was considered to be statistically significant. Continuous variables are presented as the means ± standard deviations (SDs) or medians and interquartile ranges. Categorical data are reported as frequencies and percentages. The Chi-squared test, Kruskal-Wallis test, and one-way ANOVA were used to compare the baseline characteristics of patients classified by tertile of the TG/HDL-C ratio. Survival was calculated using the Kaplan-Meier method, and differences between distributions of survival were assessed by the Breslow test. Multiple Cox proportional regression analysis was used to investigate the independent association of the TG/HDL-C ratio with mortality after adjustment for several confounders. The censored data included switching to HD, renal transplantation, moving to another center, declining additional treatment, loss to follow-up, or still attending our PD center on May 31, 2017. The fully adjusted Cox proportional regression model was adjusted for age, sex, diabetes, history of CVD, hypertension, eGFR, BMI, CCI, white blood cells, hemoglobin, platelet, serum albumin, total cholesterol, uric acid, and use of lipid-lowering agents. The results are presented as hazard ratios (HRs) ± 95% confidence intervals (CIs), and statistical significance is indicated. In sensitivity analyses, to minimize possible effects of the lipid-lowering agents, all hazard ratios were recalculated after excluding the patients who have already taken lipid-lowering medications.

## Results

### Study population

A total of 973 patients were included in this study. The baseline demographics and clinical and laboratory characteristics of the patients across tertiles of the TG/HDL-C ratio are summarized in Table 1. The mean age of the patients was 49.67 ± 14.58 years, and 57.1% were male. The percentages of hypertension, diabetes, and CVD history were 73, 19.1, and 9.9%, respectively. Patients with elevated baseline TG/HDL-C ratios compared with the reference group (T1) tended to be younger females who were more likely to have hypertension and diabetes. They also had lower serum HDL-C levels and higher BMIs and total cholesterol and TG levels.

**Table 1:** Baseline characteristics of individuals stratified by tertiles of TG/HDL-C ratio

Variables	Total	TG/HDL-C ratio			*P* value
		T1 ≤ 0.833 (*n* = 323)	0.833 < T2 ≤ 1.496 (*n* = 325)	T3 > 1.496 (*n* = 325)	
Age (y)	49.67 ± 14.58	50.95 ± 15.09	49.55 ± 14.18	48.52 ± 14.41	0.103
Male (%)	556 (57.1)	198 (61.3)	172 (52.9)	186 (57.2)	0.098
Body mass index (kg/m 2)	21.91 ± 3.34	21.17 ± 2.93	21.83 ± 3.31	22.73 ± 3.58	< 0.001
Diabetes (%)	186 (19.1)	59 (18.3)	61 (18.8)	66 (20.3)	0.789
CVD (%)	96 (9.9)	28 (8.7)	27 (8.3)	41 (12.6)	0.124
Hypertension (%)	710 (73)	223 (69)	254 (78.2)	233 (71.7)	0.027
Systolic pressure (mmHg)	146.91 ± 25.89	147.48 ± 25.57	146.96 ± 24.99	146.28 ± 27.14	0.84
Diastolic pressure (mmHg)	87.91 ± 15.89	87.34 ± 16.44	88.42 ± 14.29	87.96 ± 16.62	0.683
CCI	3.53 ± 1.88	3.60 ± 1.87	3.48 ± 1.85	3.51 ± 1.92	0.711
Total Kt/V	2.16 (1.67, 2.70)	2.15 (1.51, 2.60)	2.20 (1.73, 2.72)	2.09 (1.68, 2.74)	0.131
eGFR (ml/min per 1.73 m 2)	3.19 (1.73, 5.50)	2.90 (1.69, 4.76)	3.16 (1.71, 5.56)	3.55 (1.77, 5.82)	0.16
White blood cells (/L)	5.74 (4.50, 7.21)	5.33 (4.22, 6.90)	5.73 (4.43, 6.95)	6.15 (4.97, 7.73)	< 0.001
Hemoglobin (g/L)	78.89 ± 16.81	76.71 ± 14.83	78.96 ± 16.98	80.98 ± 19.0	0.005
Platelet (/L)	161.0 (118.0, 206.5)	151.0 (111.0, 192.0)	158.0 (112.0, 206.25)	176.0 (129.0, 220.25)	< 0.001
Albumin (g/L)	35.41 ± 5.25	35.56 ± 5.06	35.39 ± 5.41	35.28 ± 5.28	0.798
uric acid (mmol/L)	435 (347, 535)	428.5 (342.50, 520.75)	441.5 (356, 540)	439 (345, 562)	0.314
Total cholesterol (mmol/L)	4.06 (3.37, 4.85)	3.99 (3.23, 4.65)	4.04 (3.37, 4.82)	4.22 (3.49, 5.12)	0.005
Triglycerides (mmol/L)	1.27 (0.89, 1.76)	0.80 (0.63, 0.96)	1.26 (1.03, 1.48)	2.06 (1.69, 2.57)	< 0.001
HDL-C (mmol/L)	1.09 (0.90, 1.39)	1.40 (1.18, 1.69)	1.09 (0.95, 1.31)	0.88 (0.73, 1.03)	< 0.001
Lipid-lowering agents use (%)	91 (9.4)	23 (7.1)	23 (7.1)	45 (13.8)	0.003

Abbreviations: *CVD* Cardiovascular disease, *CCI* Charlson comorbidity index, *HDL-C* High-density lipoprotein cholesterol, *T* Tertile

*P <* 0.05 is considered statistically significant

### Outcomes according to the TG/HDL-C ratio tertiles

The median serum TG/HDL-C ratio was 1.11 (IQR = 0.71–1.80). During the median follow-up period of 27.2 months (IQR = 13.4–41.5 months), 229 (23.5%) patients died. Of these 229 patients, 184 individuals had data available on the primary cause of death, of which 120 (65.2%) deaths were attributed to CV mortality, 19 (10.3%) to infectious disease, 21 (11.4%) to cachexia, 3 (1.6%) to malignancy, and 21 (9.2%) to other reasons. The most common cause of CV death in this study was congestive heart failure (*n* = 69, 57.5%), followed by cerebrovascular accident (*n* = 42, 35%), unstable angina (*n* = 4, 3.3%), acute myocardial infarction (*n* = 3, 2.5%) and cardiac arrhythmia (*n* = 2, 1.7%).

### All-cause and CV mortality

**Fig. 2:**
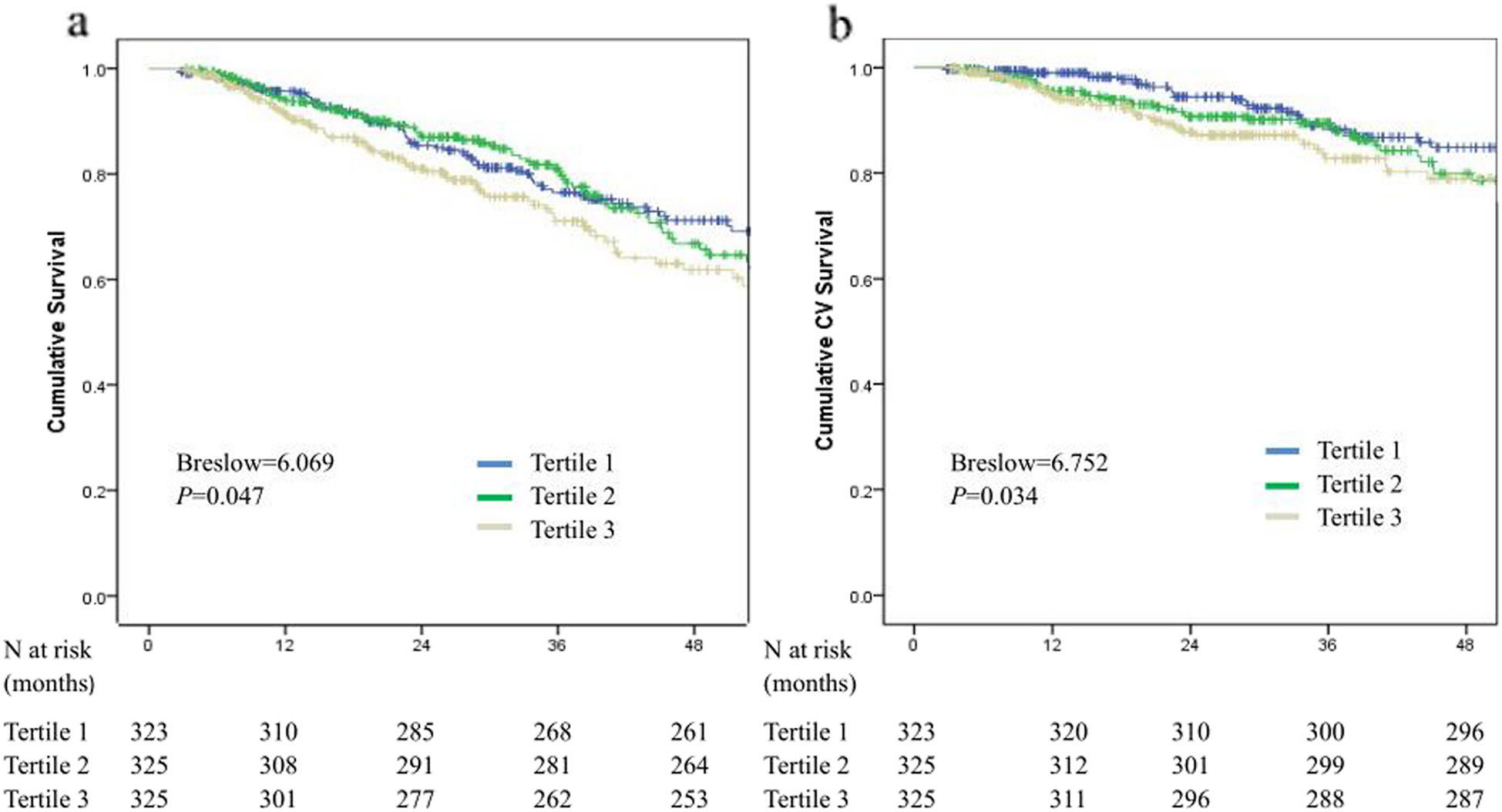
Kaplan-Meier curves for patients stratified by TG/HDL-C ratio. **a** All-cause mortality curves. **b** Cardiovascular mortality curves

Kaplan-Meier survival estimates of all-cause and CV mortality with different levels of TG/HDL-C ratios are shown in Fig. 2. Patient survival rates were lower in the T3 group than in the T1 group (*P* = 0.047) (Fig. 2a). Similarly, patients in the T3 group had the lowest CV survival rate among the groups (*P* = 0.034) (Fig. 2b). The association between the TG/HDL-C ratio and all-cause and CV mortality was determined by using Cox regression analysis. As shown in Table 2, after adjusting for age, sex, diabetes, history of CVD, hypertension, eGFR, BMI, CCI, white blood cells, hemoglobin, platelet, serum albumin, total cholesterol, uric acid and the use of lipid-lowering agents, the TG/HDL-C ratio remained associated with all-cause and CV mortality. In model 4, the HRs and 95% CIs for tertile 3 versus tertile 1 were: HR, 2.08 (95% CI: 1.37–3.14; *P* = 0.001) and HR, 2.12 (95% CI: 1.21–3.72; *P* = 0.009) for all-cause and CV mortality, respectively. In sensitivity analysis, we excluded the patients with lipid-lowering agents used, the results were materially unchanged (data shown in the supplemental file for Table 3). However, when the ratio was examined as a continuous variable, the association between the TG/HDL-C ratio and all-cause and CV mortality was not significant (HR, 1.05; 95% CI, 0.98–1.13; *P* = 0.18, HR, 1.07; 95% CI, 0.98–1.17; *P* = 0.113, for all-cause and CV mortality).

**Table 2:** The associations of TG/HDL-C ratio with all-cause mortality and cardiovascular mortality

	Tertile 2		Tertile 3	
	HR (95% CI)	*P* value	HR (95% CI)	*P* value
All-cause mortality				
Model 1	1.14 (0.83–1.57)	0.433	1.79(1.29–2.47)	< 0.001
Model 2	1.30 (0.89–1.91)	0.175	2.10 (1.41–3.14)	< 0.001
Model 3	1.23 (0.87–1.91)	0.206	2.02 (1.34–3.04)	0.001
Model 4	1.31 (0.88–1.96)	0.186	2.08 (1.37–3.14)	0.001
Cardiovascular mortality				
Model 1	1.29 (0.83–2.02)	0.262	1.90 (1.21–2.98)	0.005
Model 2	1.42 (0.83–2.42)	0.204	2.11 (1.22–3.66)	0.008
Model 3	1.43 (0.83–2.47)	0.197	2.09 (1.19–3.67)	0.01
Model 4	1.43 (0.82–2.47)	0.208	2.12 (1.21–3.72)	0.009

Model 1: adjusted for age, sex

Model 2: model 1 adjusted for diabetes, history of CVD, hypertension, eGFR, BMI, Charlson comorbidity index

Model 3: model 2 adjusted for white blood cells, hemoglobin, platelet, serum albumin, total cholesterol, uric acid

Model 4: model 3 adjusted for lipid-lowering agents use

The tertile 1 as the reference

**Table 3:** The associations of TG/HDL-C ratio with all-cause and cardiovascular mortality after excluding the patients with the lipid-lowering agents used

	Tertile 2		Tertile 3	
	HR (95% CI)	*P* value	HR (95% CI)	*P* value
All-cause mortality				
Model 1	1.11(0.79–1.56)	0.547	1.59(1.13–2.23)	0.007
Model 2	1.29(0.86–1.94)	0.215	1.83(1.20–2.77)	0.005
Model 3	1.31(0.87–1.98)	0.201	1.72(1012–2.65)	0.013
Cardiovascular mortality				
Model 1	1.20(0.74–1.94)	0.454	1.61(1.001–2.59)	0.045
Model 2	1.32 (0.74–2.35)	0.344	1.86(1.05–2.35)	0.035
Model 3	1.36(0.75–2.44)	0.308	1.85(1.02–3.6)	0.043

Model 1: adjusted for age, sex

Model 2: model 1 adjusted for diabetes, history of CVD, hypertension, eGFR, BMI, Charlson comorbidity index

Model 3: model 2 adjusted for white blood cells, hemoglobin, platelet, serum albumin, total cholesterol, uric acid

The tertile 1 as the reference

Therefore, the association between the TG/HDL-C ratio and all-cause and CV mortality in terms of sex and age was further studied using Cox regression models after adjusting for the covariates mentioned above. As shown in Table 4, with each 1-unit increase in the TG/HDL-C ratio, the adjusted HRs of all-cause and CV mortality were 1.23 (95% CI: 1.10–1.37; *P* <  0.001) and 1.24 (95% CI: 1.09–1.42-1.41; *P* = 0.001), respectively, for female patients; however, the association for male patients was not significant. In the subgroup analysis of age, for older patients (> 60 years), each 1-unit baseline increase in TG/HDL-C was associated with a 48% (95% CI: 1.06–2.07; *P* = 0.021) increased risk of all-cause mortality and a 59% (95% CI: 1.03–2.45; *P* = 0.038) increased risk of CV mortality. However, these associations were not observed in patients < 60 years of age.

**Table 4:** All-cause mortality and cardiovascular mortality for each 1-unit increase in TG/HDL-C ratio by age and sex

	Age ≤ 60 (y)^a^		Age > 60 (y)^a^		Male^b^		Female^b^	
	HR (95% CI)	*P* value	HR (95% CI)	*P* value	HR (95% CI)	*P* value	HR (95% CI)	*P* value
All-cause mortality	1.09 (0.99–1.18)	0.079	1.48 (1.06–2.07)	0.021	1.04 (0.95–1.15)	0.385	1.23 (1.10–1.37)	< 0.001
Cardiovascular mortality	1.11(0.99–1.23)	0.061	1.59(1.03–2.45)	0.038	1.05 (0.93–1.19)	0.413	1.24 (1.09–1.42)	0.001

^a^ Adjusted for sex, diabetes, history of CVD, hypertension, eGFR, BMI, Charlson comorbidity index, white blood cells, hemoglobin, platelet, serum albumin, total cholesterol, uric acid, lipid-lowering agents use

^b^ Adjusted for age, diabetes, history of CVD, hypertension, eGFR, BMI, Charlson comorbidity index, white blood cells, hemoglobin, platelet, serum albumin, total cholesterol, uric acid, lipid-lowering agents use

## Discussion

In this retrospective cohort study, we identified correlations between serum TG/HDL-C ratios and PD patient characteristics. Our two principal findings were 1) that higher serum TG/HDL-C ratios independently predicted all-cause and CV mortality in PD patients and the sensitivity analysis indicated the same findings after excluding the patients with the lipid-lowering agents used.2) subgroup analysis demonstrated that an elevated TG/HDL-C ratio was associated with higher all-cause and CV mortality in older (> 60 years) and female patients.

Our study found that higher serum TG/HDL-C ratios independently predicted all-cause and CV mortality, which are consistent with the previous studies [15, 22]. The mechanisms underlying how a higher TG/HDL-C ratio predicts a higher incidence of all-cause and CV mortality in PD patients are unclear. There are, however, some potential explanations. First, insulin resistance (IR) may play a role. There are many reports in the literature that discuss how the TG/HDL-C ratio is a useful surrogate for estimating insulin resistance (IR) [23–26]. Moreover, IR is associated with an increased risk of metabolic abnormalities, including hyperglycemia, dyslipidemia, and hypertension [27, 28], which are associated with increases in CV mortality. Importantly, Reardon et al. [29] reported that IR can promote atherogenesis through the IFNγ-macrophage pathway. Second, the higher TG/HDL-C ratio represents either increased TG or decreased HDL-C. Currently, evidence suggests that increased TG could play an important role in increased atherosclerosis [30]. However, HDL-C is heterogeneous with anti-atherogenic functions and nonvascular effects [31]. Thus, the TG/HDL-C ratio reflects a balance between atherogenic and protective lipoproteins. Moreover, dyslipidemia, characterized by high levels of TG or low levels of HDL-C, is a common risk factor for atherogenic CVD [32]. Last, oxidative stress and inflammation might be a reason. A previous study showed that the TG/HDL-C ratio was significantly associated with the presence of small and dense low density lipoprotein (LDL) cholesterol particles, which are actively taken up by arterial tissue and cause oxidative damage [33]. Accumulation of oxidized LDL cholesterol stimulates monocytes and macrophages to secrete proinflammatory cytokines and chemokines [34]. Moreover, the different food habit maybe another reason, Scicchitano P et al. [35] revealed that the food, especially the nutraceuticals, may play a peculiar role in ameliorating human dyslipidemia, and may through their antioxidant action on free radicals or by acting as anti-inflammatory molecules to decrease the incidence and prevalence of cardiovascular events. Therefore, further studies are needed to find the exact mechanisms.

In addition, our study also found that elevated TG/HDL-C ratio was associated with higher all-cause and CV mortality in female patients but not in male patients, which are consistent with the previous studies [18, 22]. The mechanism underlying is uncertain; the following may be the causes. It is well know that the TG and HDL cholesterol concentrations vary with gender, moreover, previous study showed that, in the general population, women tend to have lower TG and higher HDL-C levels than their male counterparts, so the women have lower TG/HDL-C ratio than men [36]. Therefore, maybe the dyslipidemia play a pivotal role on higher mortality in female. However, previous studies only indicated the sex-related difference between the TG/HDL-C ratio and mortality, our study extends previous findings by reporting the age-related difference between the TG/HDL-C ratio and all-cause and CV mortality. We demonstrated that an elevated TG/HDL-C ratio was associated with higher all-cause and CV mortality in older patients. We could not determine causality or the underlying mechanisms for this phenomenon due to the following potential reasons. As is known to all, throughout the world, the number of people aged 60 years or older has rapidly increased [37]. However, previous studies have indicated that older people have a high prevalence of dyslipidemia, which is an important, modifiable risk factor for CVD [38]. Moreover, low HDL-C levels have already been established as determinants of CV mortality among elderly populations. This may be due to HDL-C having anti-inflammatory, antioxidant, antiaggregant, anticoagulant and profibrinolytic properties, which promote the maintenance of endothelial functions; thus, the low HDL-C level may accelerate atherogenesis [37, 39].

Our study has several potential limitations that should be considered. First, this was a retrospective study, which can only reveal associations but not causality. Second, these results are from a single center, and the applicability of our findings to other geographical areas is limited. Third, because of the limited sample size, the potential risk factors were not all adjusted for in this cohort study. Hence, the effects of residual confounding factors cannot be completely eliminated. Our future studies will address these issues.

## Conclusions

In conclusion, our study found that a higher serum TG/HDL-C ratio was an independent predictor of all-cause and CV mortality in PD patients. Furthermore, the elevated TG/HDL-C ratio was significantly associated with higher all-cause and CV mortality in older PD patients.

## Data Availability

All data generated or analyzed during this study are included in this published article.

## Abbreviations

BMI: Body mass index
CCI: Charlson comorbidity index
CHOL: Total cholesterol
CIs: Confidence intervals
CV: Cardiovascular
CVD: Cardiovascular disease
eGFR: Estimated glomerular filtration rate
ESRD: End-stage renal diseas
HD: Hemodialysis
HDL-C: high-density lipoprotein cholesterol
HR: Hazard ratio
IR: Insulin resistance
LDL: Low density lipoprotein
PD: Peritoneal dialysis
SDs: Standard deviations
TG: Triglycerides
TG/HDL-C: Triglyceride to high-density lipoprotein cholesterol ratio
